# Urticaria and silent parasitism by Ascaridoidea: Component-resolved diagnosis reinforces the significance of this association

**DOI:** 10.1371/journal.pntd.0008177

**Published:** 2020-04-03

**Authors:** Marta Viñas, Idoia Postigo, Ester Suñén, Jorge Martínez

**Affiliations:** 1 Allergy Service, Hospital de Terrassa, Consorci Sanitari de Terrassa, Carretera de Torrebonica, Barcelona, Spain; 2 Laboratory of Parasitology and Allergy, Research Center Lascaray, Department of Immunology, Microbiology and Parasitology, University of the Basque Country, Faculty of Pharmacy, Paseo de la Universidad, Vitoria, Spain; Consejo Nacional de Investigaciones Cientificas y Tecnicas, Fundación Mundo Sano, ARGENTINA

## Abstract

Urticaria remains a major problem in terms of aetiology, investigation, and management, and although parasitic diseases are considered potential causes, the absence of a consistent link between parasitic infections and skin allergy symptoms leads to the need for a deeper study of parameters that support this association. The objectives of this study were to analyse a possible relationship between parasitism by Ascarididae (*Toxocara canis* and *Anisakis simplex*) and the clinical expression of urticaria and to identify possible parasitic molecular markers for improving the diagnosis of unknown urticaria aetiology. The prevalence of *Toxocara* and *Anisakis* infestations was evaluated by measuring the levels of specific IgG (sIgG) and IgE (sIgE) antibodies against crude extracts and isolated components from whole larvae of *Anisakis simplex* (Ani s 1, Ani s 3 and Ani s 7) and *Toxocara canis* (TES-120, TES-70, TES-32 and TES-26) using immunologic and molecular diagnostic methods. A cross-sectional study was performed in a group of 400 individuals. The study group consisted of 95 patients diagnosed with urticaria (55 with chronic urticaria and 40 with acute urticaria). A control group consisted of 305 subjects without urticaria (182 diagnosed with respiratory allergy and 123 without allergy). Statistically significant differences were demonstrated in the seroprevalence of specific IgG and IgE antibodies between the urticaria patients and the healthy general population when isolated ascarid antigens were evaluated. The prevalence of IgG antibodies against Ani s 1, IgE antibodies against TES-120 and IgE antibodies against TES-70 were significantly different between the control individuals (healthy general population) and patients with urticaria. Moreover, the urticaria patient group demonstrated a higher seroprevalence of antibodies (sIgE and sIgG) against *Anisakis simplex* larva whole extract than the control group but just with statistically diferences when sIgE was evaluated. The presence of IgE and/or IgG antibodies against Ani s 3 (tropomyosin) can help to discriminate between patients with and without urticaria. Both ascarids seem to be associated with urticaria, although in our region, *Anisakis* seems to have greater involvement than *Toxocara* in this relationship. Molecular diagnostics can be used to associate urticaria with parasite infestations. Tropomyosin and Ani s 1 were the most relevant markers to demonstrate the association between urticaria and the most relevant Ascarididae parasites in our region.

## Author summary

Urticaria remains a major problem in terms of aetiology, investigation, and management. It seems that some parasitic diseases could be causes of urticaria. Although there is no definitive consistent link between both entities, several studies have shown a significant positive association between parasite infections, mainly helminthic infections, and urticaria. The study of prevalence data for the most relevant Ascarididae infections in our region in different groups (healthy general population, respiratory allergy patients and patients with urticaria), through the measure of specific IgE and IgG antibodies against crude and molecular antigens of *Anisakis* and *Toxocara*, allowed us to define some antigenic markers that can be used to discriminate urticaria patients from the control individuals.

## Introduction

Urticaria is a disease characterized by the development of wheals (hives), angioedema, or both. The differential diagnosis includes anaphylaxis, autoinflammatory syndromes, and hereditary angioedema. Chronic spontaneous urticaria is defined as the recurrent development of transient wheals (hives), angioedema, or both for >6 weeks due to known or unknown causes [1]. Chronic urticaria remains a major problem in terms of aetiology, investigation, and management, and it is important to identify patients in whom physical urticaria is the main cause of disability [2,3].

Although parasitic diseases are considered a potential cause of urticaria, the absence of a consistent link between parasitic infections and skin allergic symptoms in clinical investigations stands in contrast to the fact that some parasites are the most potent inducers of Th2 responses that exist in nature [4,5]. Consequently, a clear association between parasitic infection and chronic urticaria should be better established.

In more than 90% of chronic spontaneous urticaria cases seen in routine clinical practices, the search for underlying causes is usually unsuccessful [1]. Nonetheless, autoimmunity, food intolerance, and bacterial, viral, fungal, or internal parasitic infections have all been described as probable causes of urticaria development [6].

It is recommended to collect “fresh stool” samples from patients with unexplained eosinophilia and/or a relevant history of travel abroad, in order to test them for the presence of cysts, ova, or other parasitic forms. Serology is recommended and can play a fundamental role in those parasitic diseases that are exceedingly difficult to diagnose directly, such as toxocariasis or anisakiasis [7]. Epidemiological surveys have shown that human toxocariasis and ascariasis are two very common helminthiases caused by species of Ascarididae, with a worldwide distribution [8]

In Spain, the ubiquitous ascarids belonging to the genera *Toxocara* and *Anisakis* are highly prevalent [8]. Spain appears to have the highest reported incidence of anisakiasis in Europe, with approximately 8,000 cases per year where marinated anchovies are recognized as the main foodborne cause [8]. The prevalence of *Anisakis* sensitization varies widely, ranging from 22% [9] to 75% [10], depending on the region under study, which indicates that human contact with *Anisakis* larvae is very common. In addition, Kolkhir et al. and Gracia-Bara et al. [6,11] found that *Anisakis* is one of the most common parasites detected in adult patients with chronic spontaneous urticaria.

The reported *Toxocara* prevalence in Spain reaches different values, ranging from 1 to more than 40% [12–14]. These differences are most likely associated with the diversity of the studied groups in each report. Portus et al., 1989 reported a *Toxocara* prevalence of 3.6% in Barcelona. Moreover, they found important differences between subjects with eosinophilia (14%) and a control group (1%) [15].

Factors that have been associated with an increased prevalence of toxocariasis include low socioeconomic level and poor environmental hygiene [13], which are possibly exacerbated by wet and warm climates, such as those in tropical regions [16,17]. Immigration from tropical regions to Europe is on the rise, with Spain as a frequent destination; this factor should be taken into account to evaluate possible urticaria symptoms due to *Toxocara* infections. Despite of this, data on the high seroprevalence of toxocariasis among immigrants from Latin America to Western countries (primarily Europe, the United States and Canada) are very scarce [18–20].

On the other hand, the results regarding whether parasite infections have a protective or predictive role for allergy and asthma are controversial [21]. It seems that low-prevalence or occasional helminthiasis, which is typical in industrialized countries, has an allergic reactivity-stimulating role, while high-prevalence parasitic infections, which are typical in developing countries, has a suppressive role [21,22].

There are many reports that recommend including the serodiagnosis of ascarids in the standard working diagnostic methodology, mainly in those patients who present with idiopathic chronic urticaria [23,24], with high environmental exposure [25] (rural areas, a high level of contact with dogs), with an atopic condition [26,27], and, in general, with a poor response to the usual treatment with antihistamines.

Since these parasitoses are silent in most cases, conducting these types of studies in a routine manner would allow a solid database based on evidence on which more efficient protocols for diagnosis could be proposed, while also highlighting the importance of establishing control programmes for these zoonoses and determining their role in the development of allergic manifestations [13,14,26].

Within this context, the aim of this study was to evaluate whether the two most common ascarids in Spain (*Toxocara* and *Anisakis*) might be involved in the development of urticaria and how the isolated antigenic/allergenic components of both genera could be associated with urticaria status. The application of component-resolved diagnosis could help define some diagnostic markers for urticaria caused by Ascarididae infections or exposure.

For this purpose, the seroprevalence of toxocariasis and anisakiasis in individuals with urticaria was measured through the presence of specific IgG (sIgG) and IgE (sIgE) antibodies against crude and isolated antigens/allergens using molecular diagnostic methods concretely Component-resolved diagnosis (CRD) [28] in comparison to that in individuals without urticaria, including healthy general population and atopic patients.

## Materials and methods

### Ethics statement

The Consorci Sanitari de Terrassa's Clinical Research Ethics Committee approved the study called: “Urticaria and silent parasitism by Ascaridoidea” to be carried out at the Terrassa’s Hospital. All participating subjects signed an informed consent to collect a blood sample and publish the results anonymously.

### Study population

A total of 2,798 adult patients (≥16 years old) who presented for the first time at the Allergy Service of Terrassa Hospital (Barcelona) during a 24-month period (from June 2009 to May 2011), were asked to participate in this study. Among them, 277 individuals who met the following inclusion criteria were enrolled.

### Inclusion criteria

Case population: Adult subjects (≥16 years old) presenting with urticaria.

Control populations: Adult subjects (≥16 years old) sensitized to common pneumoallergens and presenting with allergy symptoms, such as rhinitis and/or asthma.

A group of 123 serum samples from the healthy general population (National Register of Biobank Serum Collections, code C.0002774; Instituto de Salud Carlos III, Ministry of Economy and Competitiveness/Lascaray Research Center, University of the Basque Country, Vitoria, Spain) was also included. None of the subjects presented allergy or urticaria symptoms, according to a personal history questionnaire filled in before the blood draw.

### Clinical and epidemiological data

Clinical and epidemiological data on each subject were obtained using questionnaires designed for this purpose. The questionnaire covered age, sex, nationality, living conditions, health status, previous and current allergic manifestations, geophagia, and the presence of gastrointestinal and cutaneous symptoms, including chronic or acute urticaria, itching, and atopic dermatitis.

Other related questions, such as those regarding personal hygiene, drug use, weight loss, travel history during the last 5 years, and contact with animals, were also included in the questionnaire. The questionnaire was completed by the doctor who attended to the patients at the Allergy Service and urticaria was diagnosed following the EAACI/GA2LEN/EDF/WAO Guideline [1].

A written informed consent for diagnostic procedures and questionnaires was obtained from all patients. The study protocol was approved by the Clinical Investigation Ethics Committee of Terrassa Health Trust (Barcelona).

### Skin prick tests

All patients underwent skin prick testing [29] with a standardized battery of the predominant aeroallergenic extracts of the area where the study was conducted: mites (*Dermatophagoides pteronyssinus*, *Dermatophagoides farinae*), moulds (*Alternaria alternata*, *Cladosporium herbarum*), dog and cat epithelia and pollens (Cupresaceae, *Lolium perenne*, *Cynodon dactylon*, *Phragmites communis*, *Olea europaea*, *Platanus acerifolia*, *Chenopodium album*, *Pinus sp*., *Parietaria judaica*, *Plantago lanceolata* and *Artemisia vulgaris)*.

### Serum samples for biochemical and immunochemical analyses

A 5-mL serum aliquot was obtained from each patient for immunological and biochemical analyses. The samples were identified according to standard Spanish rules for biomedical investigations and stored at -40°C until analysis. Complete blood count, serum biochemistry tests (including the level of eosinophil cationic protein), and laboratory determination of total and specific serum IgE levels, when indicated, were performed.

### Immunological assays

#### Atopy

Atopy was analysed using the ImmunoCAP Phadiatop (Thermo Fisher Scientific, USA) assay [30], a solid-phase immunoassay for serum specific IgE antibodies against a balanced mixture of relevant inhaled allergens. The manufacturer’s protocol was followed. Because *Salsola* allergen extract is not included in the ImmunoCAP Phadiatop panel, the specific IgE antibody against this allergenic source was measured in all subjects separately with an ImmunoCAP specific IgE assay (Thermo Fisher Scientific, USA).

### Immunoanalysis using *Toxocara canis* L2 excretory-secretory (TES) whole antigens (WA)

*Quantification of the sIgG antibodies anti-Toxocara canis L2 excretory-secretory whole antigens (WATES)*. Quantification of the specific IgG antibodies against *T*. *canis* was performed by ELISA (EIA Toxocara IgG, TEST LINE Clin. Diag. Ltd., Czech Republic) according to the manufacturer’s instructions [31]. The products coupled to the solid phase were the excretory-secretory antigens from *T*. *canis* L2 larvae. The cut-off value was > 0.90 mg/ml.

*Quantification of the sIgE antibodies anti-Toxocara canis L2 excretory-secretory whole antigens (WATES)*. Quantification of the specific IgE antibodies against *T*. *canis* was performed by FEIA (ImmunoCAP, Thermo Fisher Scientific, USA). Briefly, biotinylated WATES antigens were coupled to streptavidin-activated ImmunoCAPs according to the method of Rodrigo et al. [32]. The FEIA method was performed according to the manufacturer’s instructions. The cut-off value was 0.1 kU_A_/L.

### Immunoanalysis using *Anisakis simplex* larvae whole antigens

*Quantification of the sIgG and sIgE antibodies anti-Anisakis simplex larva whole antigens*. Quantification of specific IgE and IgG antibodies against *A*. *simplex* larva crude antigenic extract was carried out by FEIA (ImmunoCAP, Thermo Fisher Scientific, USA) [32,33]. The cut-off value for sIgE was 0.10 kU_A_/L according to the manufacturer’s instructions, and for sIgG, the cut-off value was 4.3 mg/ml calculated by ROC analysis since there are no reference normal values for sIgG.

### Component-resolved diagnosis

*Quantification of sIgG and sIgE against the Anisakis simplex isolated allergens Ani s 1*, *Ani s 3 and Ani s 7*. Quantification of specific IgG and IgE antibodies against *A*. *simplex* recombinant allergens (Ani s 1 and Ani s 7) was performed by ELISA (Trisakis 170, Santiago de Compostela, Spain) [34,35,36]. The ELISA was performed according to the manufacturer’s instructions. The cut-off value for sIgE against Ani s 1 was > 0.10 kUA/L, > 0.05 kUA/L for sIgE against Ani s 7, and > 0.133 mg/ml for sIgG against Ani s 1 and Ani s 7.

Quantification of specific IgG and IgE antibodies against the *A*. *simplex* recombinant allergen Ani s 3 was performed by FEIA (ImmunoCAP, Thermo Fisher Scientific, USA). The cut-off value for sIgE was > 0.10 kUA/L and for sIgG was > 4.4 mg/ml.

*Determination of the levels of sIgG and sIgE against the TES-120*, *TES-70*, *TES-32 and TES-26 specific antigens for diagnosis of Toxocara*. Levels of sIgG and sIgE against TES-L2 antigenic extracts were analysed by immunoblotting (Immunoblot Kits Test Line Clinical Diagnostics Ltd., Czech Republic). For determining sIgG content, the manufacture´s indications were followed. To determine sIgE content, the protocol was modified according to the standard sIgE immunoblotting method used in our laboratories [37]. Briefly, serum samples were incubated while shaking overnight with immunoblotted TES antigens at 4°C. After washing (3xTBS-Tween 1%), the assay was developed with anti-human-sIgE-HRP (1:10,000 dilution) (SouthBiotech) and 4-chloronaphthol.

All immunological tests were performed blindly by the study group, with samples identified only by patient codes. The tests were developed by the same investigator at the Parasitology and Immunoallergy Laboratory of the Lascaray Research Center in Vitoria.

### Evaluation of results

Quantitative variables (results of specific IgE and IgG content) were requalified into qualitative variables. The values of the variables that were equal to or greater than the cut-off point were considered positive and the values lower than the cut-off were considered negative. Therefore, 2 qualitative variables were always compared.

### Statistical analysis

Qualitative variables are expressed as absolute frequencies and percentages, and quantitative variables are expressed as the mean ± standard deviation. Comparisons between qualitative variables were performed using the Pearson chi-square test and Fisher’s exact test with 2x2 tables. Statistical significance was accepted at p≤0.05.

All data of interest were entered into an Excel 2003 for Windows database (Microsoft Corp., Redmond, WA, USA) by a single investigator. Statistical analysis was then performed in IBM SPSS Statistics for Windows, version 21.0 (IBM Corp., Armonk, NY, USA). Analysis of *Anisakis*-specific IgG antibodies was performed using GraphPad Prism version 7.03 for Windows. To calculate the cut-off value for the specific anti-*Anisakis* IgG value measured by FEIA ImmunoCAP, a receiver operating characteristic (ROC) curve was constructed.

## Results

Table 1 shows the clinical diagnosis distribution in the 400 individuals included in the study (60% female, 40% male; mean age: 38.5 ± 12.5 years). A total of 23.75% of the studied population was diagnosed with urticaria (95/400). According to symptom persistence, 13.75% of the subjects (55/400) suffered from chronic urticaria, and 10% suffered from acute urticaria (40/400). A total of 58.25% of the patients included in the study (233/400) were diagnosed with allergy, 12.75% (51/400) suffered from allergy and urticaria, and 45.50% (182/400) suffered from allergy without urticaria. One hundred twenty-three subjects (30.75%) were considered healthy members of the general population. The individuals belonging to this group had no symptoms of allergy or urticaria, and 32% of them were atopic coinciding with the reported prevalence in the Spanish population [38].

**Table 1:** Clinical group distribution of the total sample population (n = 400).

	No urticaria		Chronic urticaria		Acute urticaria	
	N	%	N	%	N	%
**Allergic subjects**	**182**	**45.50**	**23**	**5.75**	**28**	**7.00**
**Non allergic subjects**	**123**	**30.75**	**32**	**8.00**	**12**	**3.00**
**TOTAL**	**305**	**76.25**	**55**	**13.75**	**40**	**10.00**

Reference normal values for the specific anti-*Anisakis* IgG level measured by FEIA (ImmunoCAP) was not previously established. Therefore, in order to establish a cut-off value that would allow the calculation of the seroprevalence of this antibody class in the study population, the distribution of this value was analysed in all three groups of the study: healthy general population, patients with urticaria, and patients without urticaria.

**Fig 1:**
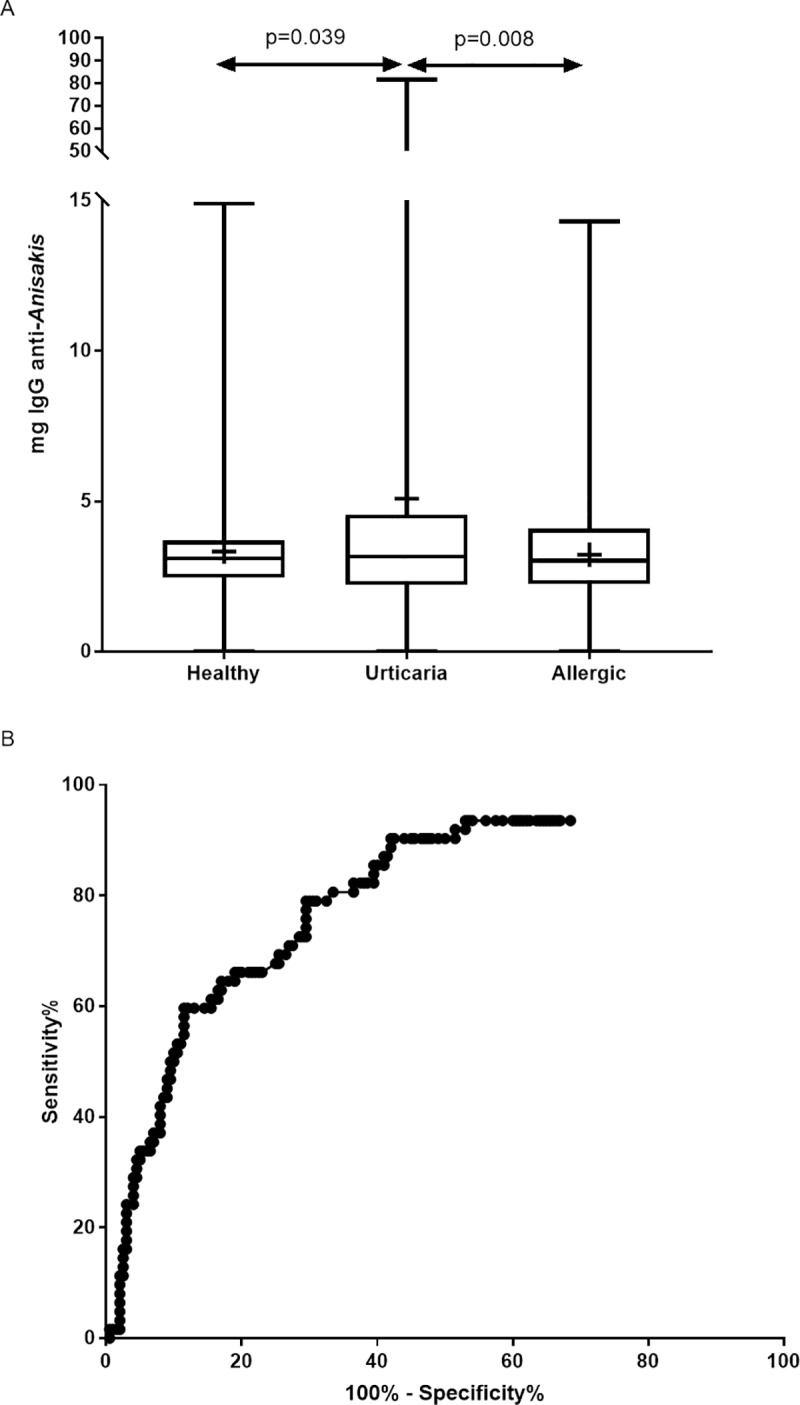
(A) Statistical analysis of specific IgG to Anisakis larval antigens in the different groups of individuals included in the study: calculation of cut-off values. Box-and-whisker plot: distribution of specific IgG to Anisakis in healthy donors, urticaria, and allergic groups. (B) Receiver operating characteristic curve for calculation of the optimal cut-off point for specific anti-*Anisakis* IgG.

Fig 1A shows a box-and-whisker plot of the distribution of specific anti-*Anisakis* IgG values in these three groups. The mean values (mg/ml) and their corresponding standard deviations were 3.32 ± 1.96 in the healthy general population, 5.10 ± 10.20 in patients with urticaria, and 3.22 ± 2.30 in patients without urticaria. Statistically significant differences were found between the urticaria patient group and the control groups: subjects without urticaria (p = 0.008) and the healthy general population (p = 0.039).

The optimal cut-off point for specific IgG against *Anisakis* whole larval antigen was obtained by ROC analysis (area under the curve: 0.804; standard error: 0.032; 99% confidence interval: 0.721 to 0.886; p<0.001) (Fig 1B). The mean value plus 0.5 SD (4.3 mg/ml) was chosen as the cut-off value (sensitivity, 60%; specificity, 88%).

Table 2 shows the seroprevalence (sIgE and sIgG) of *Toxocara* and *Anisakis* sensitization in the studied population. The analysis was conducted against the *Anisakis* and *Toxocara* larva whole antigens as well as against the isolated antigenic components Ani s1, Ani s 3 and Ani s 7 and TES-120, TES-70, TES-32 and TES-26 (component-resolved diagnosis [39,40]).

**Table 2:** Seroprevalence (%) of specific IgG and IgE antibodies against *Toxocara* and *Anisakis* whole larva extract (WE) and isolated antigens in the groups included in the study.

		No urticaria		Urticaria		
		Healthy	Allergic	Total (n = 95)	Chronic (n = 55)	Acute (n = 40)
		(n = 123)	(n = 182)			
*Anisakis* (WE)	IgE	4.00	14.80	33.70	30.90	35.70
	IgG	13.00	17.00	28.40	32.70	22.50
*Toxocara* (WE)	IgE	6.50	7.10	11.60	10.90	12.50
	IgG	3.30	5.50	5.30	3.60	7.40
Ani s 1	IgE	1.60	4.40	7.40	7.30	7.50
	IgG	3.20	9.90	11.60	12.70	10.00
Ani s 3	IgE	0.25	2.14	12.60	7.60	5.00
	IgG	0.50	8.58	25.20	14.70	10.20
Ani s 7	IgE	4.30	8.50	10.50	5.40	17.50
	IgG	1.30	7.30	6.30	3.60	10.00
TES-120	IgE	1.60	2.20	4.00	3.60	5.00
	IgG	0.80	3.30	2.00	1.80	2.50
TES-70	IgE	0.40	1.10	4.10	3.60	5.00
	IgG	0	1.10	0	0	0
TES-32	IgE	0.80	0.50	1.00	0	2.50
	IgG	0	2.20	1.00	0	2.50
TES-26	IgE	0.80	0.50	1.00	0	2.50
	IgG	0.80	2.20	1.00	0	2.50
sIgE and/or sIgG against Ascaride antigens		22.70	39.00	53.60	49.00	60.00

The seroprevalence of ascarid sensitization (positive results for specific IgG and/or IgE antibodies against *Anisakis* and/or *Toxocara* antigens) was 22.70% in the healthy general population, 39% in patients suffering from allergy without urticaria and 53.60% in patients with urticaria. The highest seroprevalence was observed in patients with acute urticaria (60%).

Among the urticaria patients, the highest prevalence was demonstrated for IgG and IgE antibodies against *Anisakis* whole extract. When the analysis using the isolated antigens from both ascarids was performed, the specific IgG antibodies followed by the specific IgE antibodies against Ani s 3 demonstrated the highest prevalence among urticaria patients (25.20 and 12.60%, respectively). The seroprevalence of both antibodies in the healthy general population was very low (0.50 and 0.25%, respectively) and slightly higher in the allergic control group (8.58 and 2.14%, respectively).

These data suggests that *Anisakis* and/or *Toxocara* whole or isolated antigens could be used as markers in the diagnosis of urticaria caused by Ascarididae infections/exposure. With this result in mind, a statistical analysis (Pearson chi-square and Fisher exact test) was performed, and P values were obtained among the different groups included in the study (Table 3).

**Table 3:** Comparative analysis of the specific IgG and IgE seroprevalences among the different study groups. Results are shown as P values.

		Urticaria *vs*		Chronic urticaria *vs*		Acute urticaria *vs*	
		Healthy	Allergy without urticaria	Healthy	Allergy without urticaria	Healthy	Allergy without urticaria
*Anisakis* (WE)	IgE	0.000***	0.001***	0.000***	0.010**	0.000***	0.003**
	IgG	0.006**	0.050	0.003***	0.021**	0.204	0.495
*Toxocara* (WE)	IgE	0.229	0.261	0.369	0.397	0.310	0.332
	IgG	0.414	0.494	1.000	0.738	0.364	0.708
Ani s 1	IgE	0.043*	0.401	0.074	0.481	0.095	0.422
	IgG	0.028*	0.682	0.037*	0.616	0.104	1.000
Ani s 3	IgE	0.000***	0.000***	0.018**	0.005**	0.308	0.208
	IgG	0.000***	0.009**	0.025*	0.551	0.000***	0.139
Ani s 7	IgE	0.689	0.800	1.000	0.740	0.239	0.224
	IgG	0.385	1.000	0.149	0.475	0.669	0.727
TES-120	IgE	0.023*	0.143	0.058	0.392	0.013**	0.058
	IgG	0.045*	0.379	0.058	0.702	0.046*	0.392
TES-70	IgE	0.015**	0.049	0.094	0.231	0.014**	0.042*
	IgG	0.189	1.000	0.309	0.329	0.245	0.551
TES-32	IgE	0.436	1.000	N	1.000	0.245	0.451
	IgG	0.170	0.498	0.524	1.000	0.245	0.158
TES-26	IgE	0.189	0.609	N	1.000	0.059	0.150
	IgG	0.170	0.498	0.524	1.000	0.046*	0.158
sIgE and/or sIgG against Ascaride antigens		≤0.001***	0.397	≤0.001***	0.212	≤0.001***	0.021*

*** indicates p≤0.001

** indicates p = 0.002–0.020 and

* indicates p = 0.020–0.05

When both antibody isotypes (IgG and/or IgE) against the whole or isolated larval antigen from the ascarids *Toxocara* and *Anisakis* were evaluated, statistically significant differences were demonstrated between the healthy general population controls and the subjects suffering from urticaria (p≤0.001). No statistically significant differences were demonstrated between allergic patients without urticaria (control group) and the individuals with urticaria. Nonetheless, differences between allergic patients without urticaria and patients with acute urticaria were significant (p = 0.021).

When each antibody isotype was analysed regarding the antigenic source (whole larval antigens or isolated antigens), specific IgE antibodies against *Anisakis* larval whole extract were able to discriminate urticaria patients (chronic and acute) from the subjects without urticaria (the healthy general population and allergic patients without urticaria). Moreover, specific IgG antibodies against *Anisakis* larval whole extract were able to discriminate patients with chronic urticaria from the healthy general population (p = 0.003). On the other hand, none of the antibody isotypes against *Toxocara* larval whole antigens were able to discriminate between the controls and the urticaria (chronic or acute) patients.

Although statistically significant differences in the seroprevalence of specific IgE and IgG antibodies against Ani s 1 between urticaria patients and the healthy general population were demonstrated, only the specific IgG antibody level was able to discriminate these groups (p = 0.028). No statistically significant differences were demonstrated between the prevalence of both antibodies in urticaria patients and in the allergic patient controls.

Furthermore, both IgE and IgG antibodies against Ani s 3 were able to discriminate the chronic and acute urticaria patients from the healthy general population and from the allergic patient controls (p<0.001). These results, together with the seroprevalence data, make Ani s 3 a candidate diagnostic marker of urticaria of unknown origin. In the case of Ani s 7, seroprevalence data for specific IgG or specific IgE did not reveal significant differences. Neither IgG nor IgE antibodies were able to discriminate between the studied groups. Among the *Toxocara* isolated antigens explored as urticaria markers, TES-70 and TES-120 were able to discriminate between acute urticaria patients and the healthy general population via specific IgE antibody levels (p = 0.014 and p = 0.013, respectively).

## Discussion

Several authors agree that parasite infections are relatively common in patients with urticaria, but a causal link is difficult to detect. Such infections should only be regarded as the underlying cause of urticaria when treatment with anti-parasitc drugs leads both to the eradication of the parasites and the remission of urticaria.

Although routine screening for parasitic infections in patients with urticaria is not recommended, some authors [6,41] suggest that a parasitological workup may be warranted, depending of patient’s characteristics, such associated symptoms, dietary habits, country of origin and travel history.

In Europe, mainly in southern countries, anisakiasis [8] and toxocariasis [13] are the two most prevalent parasitic diseases caused by Ascaridoidea species in adults [14,15,26]. Both species are difficult to identify by direct parasitological methods. Therefore, indirect immunologic diagnosis is currently the most common strategy to determine the aetiology. In many cases, the epidemiological study of toxocariasis is limited by several factors that inhibit the assessment of the public health relevance of this parasitic infection [40].

However, to date, serological assays using *Toxocara* ES-L2 antigens or their recombinant components are the main tool for the diagnosis of toxocariasis [42]. Studies on the seroprevalence of toxocariasis in Spain have reported heterogeneous results (from 1 to >40%) depending on region, socioeconomic status, rural or urban environment, and pet ownership, among other factors. Spain-neighbouring countries show similar toxocariasis results [43,44], but in urban areas, the prevalence does not usually exceed 3%. In 2011, Turrientes et al. [44], demonstrated that the prevalence of toxocariasis in immigrants arriving in Spain was as high as 5%. They suggested that these subjects may have been infected in their countries of origin, mostly in Latin America, where prevalence rates are usually higher than those in Spain [45–47].

In the present study, the seroprevalence of IgG antibodies against *Toxocara* L2-ES whole extract was 3% in the healthy general population and 5.50% in allergic subjects without urticaria. When specific IgE antibodies were measured, the prevalence in the control and allergic groups was 6.50% and 7.10%, respectively. No statistically significant differences were found between these groups regardless of the isotype of immunoglobulin measured. These results agree with the data obtained by other authors in studies performed in similar populations and in the same country (Spain) [14,15,44]. Thus, in Barcelona, the same region where our study was carried out, Portús and colleagues [15] found a seroprevalence of 3.60%, with values of 14% in individuals with eosinophilia and 1% in controls.

Undoubtedly, the seroprevalence data measured by specific IgE and by specific IgG antibodies are more appropriate and more reflective of the real-world prevalence than data for a single parameter. In the literature, the prevalence of these parasitic infections, have been reported mainly through the use of only one parameter, i.e., specific IgG antibodies against toxocariasis or specific IgE antibodies against anisakiasis [43,48,49].

Both IgE and IgG antibodies play important roles not only as key parameters in immunological diagnoses but also as factors in several aspects of immunity, including protective strategies [48], and in assessing the clinical status of these parasitoses [43,49,50].

About *Toxocara*, the evaluation of both specific antibodies isotypes to ES-L2 whole antigens, did not show statistically significant differences among the studied groups, demonstrating similar toxocariasis low prevalence in all groups.

Unlike *Toxocara*, the seroprevalence of anisakiasis in Spain is quite high because is one of the European countries with the highest levels of fish consumption [49,51–55]. In the general asymptomatic population, the prevalence of sensitization to *Anisakis* antigens ranges from 0.40 to 27.40% [49]. Among occupationally exposed workers, such as fishermen, fishmongers, and workers in fish-processing industries, the presence of specific IgE antibodies against *Anisakis* larval whole extracts is documented in the range from 11.70% to 50% and between 50% to 100% when the allergy is exclusively associated to fish or seafood [49].

The results obtained in this work showed a 4% seroprevalence value for specific IgE antibodies in the healthy general population and 15% in allergic subjects without urticaria. This last group included patients with respiratory allergies, with mites the main cause of sensitization (95%). Lin et al. (2014) [55] and Caballero et al. (2012) [56] also studied the seroprevalence of specific IgE against *Anisakis* in atopic subjects and found very similar results to those reported herein (16% and 18%, respectively) [55,56]. In Italy, Qualizza et al. (2011) [57] found a low seroprevalence anisakiasis in allergic patients, with values very similar to those found in the present study in patients with urticaria. Frezzolini et al. (2010) [58] also found a similar distribution of *Anisakis* seroprevalence according to specific IgE measurements in atopic patients and in patients with chronic urticaria.

In the present study, the most significant differences in IgE antibodies against *Anisakis* larval whole extracts were observed between the healthy general population (4%) and patients with urticaria (33.70%). The group of allergic patients also showed significant differences from the subjects with urticaria (p = 0.001).

The use of specific IgG antibodies to assess the anisakiasis seroprevalence and its possible association with other diseases, such as urticaria [50], Crohn’s disease, gastrointestinal cancer, and others [59], has been reported in very few studies, and reference values for specific IgG antibodies against *Anisakis* antigens had not yet been defined. In our study, the establishment of normal values of specific IgG to *Anisakis* allowed to discriminate between individuals without urticaria and patients with urticaria, with special mention to the patients with the chronic urticaria. However, differences among all groups were clearly significant when the specific IgE antibodies against *Anisakis* larval whole extract were measured.

Nevertheless, the overall seropositivity, including both immunoglobulin isotypes against both ascarid whole antigens, was clearly higher in all studied groups and statistical significant differences were found between the healthy general population and patients with chronic or acute urticaria.

In recent decades, several isolated components from parasitic sources have emerged for diagnostic use. Antigens/allergens with diagnostic and prognostic value have been identified both in *Anisakis* and in *Toxocara* [48,50,60].

Several authors report that *Anisakis* components are used mainly in the molecular diagnosis of the allergy to *Anisakis*, and *Toxocara* components have been used mainly as tools for toxocariasis seroprevalence studies [48,50,60]. Few studies have investigated the diagnostic value of these components in urticaria, a condition in which these parasites could be involved [48–50,60–63]. In the present study, the Ani s 1, Ani s 3, TES-120 and TES-70 components were able to discriminate the healthy general population from urticaria patients.

In these cases, specific IgG against Ani s 1, specific IgE against TES-120 and specific IgE against TES-70 were useful and effective tools to discriminate the healthy general population from patients with urticaria.

Data on anti-Ani s 1 specific IgE from the literature are restricted to mainly healthy subjects or allergic patients, and the prevalence shown in these studies is similar to that obtained in the present investigation [55,64]. Martínez Aranguren et al. (2014) studied the antibody responses against Ani s 1 and tropomyosin and suggested that the use of recombinant versions makes it possible to distinguish between patients allergic to *A*. *simplex* and patients with acute urticaria who are merely sensitized to *A*. *simplex* [63].

The results obtained in this report, demonstrated that *Anisakis* tropomyosin was revealed as a very potent discriminative urticaria marker. Both specific IgE and specific IgG antibodies against this component were able to discriminate groups of individuals without urticaria from patients with urticaria. Moreover, the specific anti-tropomyosin IgG antibodies were also able to discriminate between the healthy population and patients with chronic or acute urticaria while specific anti-Ani s 3 IgE was able to discriminate the healthy population and allergic population from chronic urticaria patients. These results indicate that tropomyosin could be an interesting marker of urticaria associated with ascarid infections in our region.

If we accept that tropomyosin-specific IgE but not IgG antibodies play an important role in the sensitization of allergic patients, the use of both parameters could be interesting to differentiate sensitization from actual parasitic infection.

The results obtained in this work using diagnosis by components based on the Ani s 1, TES-120 and TES-70 antigens, strongly suggest that the molecular diagnosis is a useful tool for discriminating the healthy population from urticaria patients in our region.

Immunological cross reactions among different ascarids and among ascarids and mites or crustaceans have been demonstrated by several authors [65–69]. On the other hand, it has been shown that most detectable specific IgE is due to previous subclinical parasitic episodes with *A*. *simplex* in subjects exposed due to fish-eating habits [50,70]. Additionally, immunodiagnosis for toxocariasis reveals cross-reactivity with other nematode infections, and the positive results indicating this infection should be evaluated very carefully, especially in individuals from regions where polyparasitism is endemic or where the incidence of different parasitic nematode species in the same area is high [19]. Immunological diagnostic techinques for toxocariasis and anisakiasis are often based on the use of whole larval extracts, and the cross-reactivity phenomenon is particularly evident under these conditions. Limitations of results when whole antigens are used must be taken into account.

In our area, polyparasitism by different nematodes or the incidence of nematodes other than *Toxocara* or *Anisakis* is highly irrelevant. Therefore, only the cross-reactive antigens/allergens shared not only with other helminths but also with mites or crustaceans should be taken into account when establishing the relationship between parasitism by ascarids and urticaria. In recent years, with the use of isolated components, the concept of cross-reactivity has been reviewed, and the concept of species-specific components and cross-reactive antigens/allergens is becoming more accurate and much better delimited [51,71,72].

Investigations of Spanish and Italian patients allergic to *Anisakis* demonstrated that Ani s 1 is the immunodominant specific allergen, and the quantification of IgE specific to Ani s 1 is able to discriminate between asymptomatic and allergic patients [51,63]. Several authors have also demonstrated that some lectins, such as TES-32, are immunodominant components for diagnostic purposes [73,74]. Yamasaki and colleagues reported an increase in specificity when they used the recombinant antigen in comparison with larval crude extract. They found a decreasing percentage of cross-reactivity from 55% to 3% when the recombinant antigen was used [75].

Although cross-reactivity can be a limitation to establish the association between the studied ascarids and urticaria, mainly when larval crude extracts are used, the differences in prevalence among the urticaria patients and the reference groups found in this study are relevant enough to alleviate this limitation. This point is even more evident if we take into account the *Anisakis* results using mainly specific immunodominant components through IgE quantification.

Surprisingly, in our country, the panallergen tropomyosin is the component that is most significant for studying the association between ascarids, represented mainly by *Anisaki*s and urticaria. Cross-reactions with mite allergens or shellfish allergens could also be a limitation to explain this association. The results obtained in this work show that the prevalence of tropomyosin for the allergy patients was significant lower than the obtained for the urticaria group. The differences were very significant and more so if we consider that 40% of the allergic patients were sensitized to house dust mites. A similar concept could be applied to the limitations due to shellfish allergy. Nwaru and colleagues reported a very low prevalence of shellfish allergy in Europe, with values from 0.06% and 0.10% [76].

Although both *Toxocara* and *Anisakis* seem to be involved in the association between parasitism and urticaria, in our region, the genus *Anisakis* appears to be the most important member of this taxonomic group regarding this association. The prevalence of anisakiasis was higher than that of toxocariasis, as was expected, as dietary habits determine the high prevalence of anisakiasis in this population.

Despite the apparent limitations to establish the ascarid-urticaria association due to mite and shellfish sensitization or other helminth infections in our region, the results obtained in this work, where helminth poly-parasitism is very scarce, strongly suggest that specific IgE and IgG antibodies against *Anisakis* larva crude extract, Ani s 1 and tropomyosin could be considered markers for parasite-caused urticaria. The low prevalence of *Toxocara* found in this study and its significance regarding the healthy general population and urticaria patients make this species a smaller factor, but it should not be excluded as a possible cause of urticaria.

The use of different antibodies isotypes and the component resolved diagnosis concept are useful to establish the association between parasite immunologic results and patients with different clinic status as allergy, acute urticaria and chronic urticaria.

## Supporting information

S1 Checklist STROBE checklist. (DOCX)
