# Supplementary material for: Urticaria and silent parasitism by Ascaridoidea: Component-resolved diagnosis reinforces the significance of this association
Source: PLoS Negl Trop Dis. 2020 Apr 3;14(4):e0008177. doi: 10.1371/journal.pntd.0008177 (PMC7170265; doi:10.1371/journal.pntd.0008177)
Supplement: S1 Checklist — (DOCX) [file pntd.0008177.s001.docx]

STROBE Statement—checklist of items that should be included in reports of observational studies

|  | Item No. | Recommendation | Page  No. | Relevant text from manuscript |
| --- | --- | --- | --- | --- |
| **Title and abstract** | 1 | (*a*) Indicate the study’s design with a commonly used term in the title or the abstract | 1 paragrah 1 |  |
|  |  | (*b*) Provide in the abstract an informative and balanced summary of what was done and what was found | page 3: paragrahs 3,4, 5 and page 4 paragrah 1 |  |
| Introduction | | | |  |
| Background/rationale | 2 | Explain the scientific background and rationale for the investigation being reported | 5 paragrahs 3,4  Page 6 paragrah 2 and page 7: paragraph 1, page 8 paragrah 1 |  |
| Objectives | 3 | State specific objectives, including any prespecified hypotheses | 8  Paragrahs 2,3 | to evaluate whether the two most common ascarids in Spain (Toxocara and Anisakis) might be involved in the development of urticaria and how the individualized antigenic/allergenic components could be associated with the urticaria status, as well as to try to define some diagnostic markers for urticaria caused by Ascaridoidea infestations. |
| Methods | | | |  |
| Study design | 4 | Present key elements of study design early in the paper | 9  Paragraph 1 | to evaluate the individualized antigenic/allergenic for ascarids |
| Setting | 5 | Describe the setting, locations, and relevant dates, including periods of recruitment, exposure, follow-up, and data collection | 9  Paragraph 1 |  |
| Participants | 6 | (*a*) *Cohort study*—Give the eligibility criteria, and the sources and methods of selection of participants. Describe methods of follow-up  *Case-control study*—Give the eligibility criteria, and the sources and methods of case ascertainment and control selection. Give the rationale for the choice of cases and controls  *Cross-sectional study*—Give the eligibility criteria, and the sources and methods of selection of participants | 9  Paragraphs 2 , 3, 4  Page 10 Paragraphs 1, 2, 3 |  |
|  |  | (*b*) *Cohort study*—For matched studies, give matching criteria and number of exposed and unexposed  *Case-control study*—For matched studies, give matching criteria and the number of controls per case |  |  |
| Variables | 7 | Clearly define all outcomes, exposures, predictors, potential confounders, and effect modifiers. Give diagnostic criteria, if applicable | 9  Paragraphs 2 , 3, 4  Page 10 Paragraphs 1, 2, 3 |  |
| Data sources/ measurement | 8* | For each variable of interest, give sources of data and details of methods of assessment (measurement). Describe comparability of assessment methods if there is more than one group | 10 Paragrah 4, page 11 paragrah 3  Page 12 paragrahs 2,3 page 13 paragrahs 2,3 |  |
| Bias | 9 | Describe any efforts to address potential sources of bias | 11  Paragraph 3 | Because Salsola allergen extract is not included in the ImmunoCAP Phadiatop panel, the specific IgE antibody against this allergenic source was measured in all subjects separately with an ImmunoCAP-specific IgE assay (Thermo Fisher Scientific, USA). |
| Study size | 10 | Explain how the study size was arrived at | 9  Paragrah 1 |  |

Continued on next page

| Quantitative variables | 11 | Explain how quantitative variables were handled in the analyses. If applicable, describe which groupings were chosen and why | No applicable |  |
| --- | --- | --- | --- | --- |
| Statistical methods | 12 | (*a*) Describe all statistical methods, including those used to control for confounding | 14 Paragrahs 2,3 |  |
|  |  | (*b*) Describe any methods used to examine subgroups and interactions | 14  Paragrah 2 |  |
|  |  | (*c*) Explain how missing data were addressed |  |  |
|  |  | (*d*) *Cohort study*—If applicable, explain how loss to follow-up was addressed  *Case-control study*—If applicable, explain how matching of cases and controls was addressed  *Cross-sectional study*—If applicable, describe analytical methods taking account of sampling strategy | 14  Paragrah 3 |  |
|  |  | (*e*) Describe any sensitivity analyses |  |  |
| Results | | | | |
| Participants | 13* | (a) Report numbers of individuals at each stage of study—eg numbers potentially eligible, examined for eligibility, confirmed eligible, included in the study, completing follow-up, and analysed | 15  paragrah 1 |  |
|  |  | (b) Give reasons for non-participation at each stage |  |  |
|  |  | (c) Consider use of a flow diagram |  |  |
| Descriptive data | 14* | (a) Give characteristics of study participants (eg demographic, clinical, social) and information on exposures and potential confounders | 15  Paragrah 2 | Table 1 |
|  |  | (b) Indicate number of participants with missing data for each variable of interest |  |  |
|  |  | (c) *Cohort study*—Summarise follow-up time (eg, average and total amount) |  |  |
| Outcome data | 15* | *Cohort study*—Report numbers of outcome events or summary measures over time |  |  |
|  |  | *Case-control study—*Report numbers in each exposure category, or summary measures of exposure |  |  |
|  |  | *Cross-sectional study—*Report numbers of outcome events or summary measures | 18  Paragrah 2  Page 19 paragrah 1 | Anisakis and/or Toxocara whole or isolated antigens could be used as markers in the diagnosis of urticaria caused by Ascaridoidea infestations. |
| Main results | 16 | (*a*) Give unadjusted estimates and, if applicable, confounder-adjusted estimates and their precision (eg, 95% confidence interval). Make clear which confounders were adjusted for and why they were included | No applicable |  |
|  |  | (*b*) Report category boundaries when continuous variables were categorized | No applicable |  |
|  |  | (*c*) If relevant, consider translating estimates of relative risk into absolute risk for a meaningful time period | No applicable |  |

Continued on next page

| Other analyses | 17 | Report other analyses done—eg analyses of subgroups and interactions, and sensitivity analyses | 17  Paragrah 2 | The mean value plus 0.5 SD (4.3 mg/mL) was chosen as the cut-off value to discriminate between positive and negative results (sensitivity, 60%; specificity, 88%). |
| --- | --- | --- | --- | --- |
| Discussion | | | | |
| Key results | 18 | Summarise key results with reference to study objectives | 23  Paragrah 4  Page 24  Paragraph 3 | In the present study, the seroprevalence of IgG antibodies against Toxocara was 3% in the healthy general population and 5.5% in allergic subjects without urticaria. When specific IgE antibodies were measured, the prevalence in the control groups was 6.5% and 7.1%, respectively. The results obtained for *Anisakis* in this work showed a 4% seroprevalence value for specific IgE antibodies in the healthy general population and 15% in allergic subjects without urticaria. |
| Limitations | 19 | Discuss limitations of the study, taking into account sources of potential bias or imprecision. Discuss both direction and magnitude of any potential bias | 29 paragrah 3, page 30 paragrah 2  Page 31 paragrah s 1,3 |  |
| Interpretation | 20 | Give a cautious overall interpretation of results considering objectives, limitations, multiplicity of analyses, results from similar studies, and other relevant evidence | 31  paragrah 3 | Despite the apparent limitations to establish the ascarid-urticaria association due to mite and shellfish sensitization or other helminth infections in our region, the results obtained in this work strongly suggest that specific IgE and IgG antibodies against Anisakis larva crude extract, Ani s 1 and tropomyosin could be considered markers for parasite-caused urticaria. The low prevalence of Toxocara found in this study and its significance regarding the healthy general population and urticaria patients make this species a smaller factor, but it should not be excluded as a possible cause of urticaria. |
| Generalisability | 21 | Discuss the generalisability (external validity) of the study results | 28  Paragrahs 6,7  Page 29 Paragrahs 1,2 | larval antigens from Anisakis simplex and Toxocara canis are useful for the study of the involvement of these ascarid parasites in urticaria in our region. The most significant differences between controls and patients with urticaria were demonstrated when specific IgE antibodies against Anisakis larval crude antigens were assessed. Specific IgG antibodies against Anisakis larval crude extract were useful for discriminating the healthy general population and allergic patients from patients with chronic urticaria.  The high seroprevalence of both antibody isotypes against the ascarid whole antigens in all groups and the statistically significant differences demonstrated among the studied groups reinforce the implication of both ascarid species in the development of urticaria.  The component-resolved diagnosis based on the Ani s1, TES-120 and TES-70 antigen/allergens of these parasites has been demonstrated to be a useful tool for discriminating the healthy population from urticaria patients.  Tropomyosin has been revealed to be a potent discriminating marker in urticaria. |
| Other information | |  | | |
| Funding | 22 | Give the source of funding and the role of the funders for the present study and, if applicable, for the original study on which the present article is based | No applicable |  |

*Give information separately for cases and controls in case-control studies and, if applicable, for exposed and unexposed groups in cohort and cross-sectional studies.

**Note:** An Explanation and Elaboration article discusses each checklist item and gives methodological background and published examples of transparent reporting. The STROBE checklist is best used in conjunction with this article (freely available on the Web sites of PLoS Medicine at http://www.plosmedicine.org/, Annals of Internal Medicine at http://www.annals.org/, and Epidemiology at http://www.epidem.com/). Information on the STROBE Initiative is available at www.strobe-statement.org.
